# Supplementary material for: Real-World Data on Chronic Myelomonocytic Leukemia: Clinical and Molecular Characteristics, Treatment, Emerging Drugs, and Patient Outcomes
Source: Cancers (Basel). 2022 Aug 25;14(17):4107. doi: 10.3390/cancers14174107 (PMC9455040; doi:10.3390/cancers14174107)
Supplement: Supplementary file 1 [file cancers-14-04107-s001.zip › cancers-1858230-supplementary.pdf]

Real-world data on chronic myelomonocytic leukemia: clinical and molecular characteristics, treatment, emerging drugs, and patient outcomes

Sandra Castaño-Díez, Mónica López-Guerra, Cristina Bosch-Castañeda, Alex Bataller, Paola Charry, Daniel Esteban, Francesca Guijarro, Carlos Jiménez-Vicente, Carlos Castillo-Girón, Albert Cortes, Alexandra Martínez-Roca, Ana Triguero, José Ramón Álamo, Silvia Beà, Dolors Costa, Dolors Colomer, María Rozman, Jordi Esteve, Marina Díaz-Beyá

Supplementary Information

Table S1: NGS panels used for gene mutation analysis.

Table S2: Cytogenetic abnormalities

Figure S1: Alluvial plot of the patients included in the study according to CPSS and CPSS-Mol scores.

Figure S2: Overall survival according to the number of mutations detected at CMML diagnosis.

Figure S3: Overall survival according to the mutational status of transcription factors.

Table S1: NGS panels used for gene mutation analysis.

**NGS panel**	Genes and regions studied
**Ion AmpliseqTM AML Research Panel** (ThermoFisher Scientific)	Coding regions: *CEBPA, DNMT3A, GATA2, TET2, TP53*<br><br>Hotspot regions: *ASXL1, BRAF, CBL, FLT3, IDH1, IDH2, JAK2, KIT, KRAS, NPM1, NRAS, PTPN11, RUNX1, WT1*
**OncomineTM Myeloid Research Panel** (ThermoFisher Scientific)	Coding regions: *ASXL1, BCOR, CALR, CEBPA, ETV6, EZH2, IKZF1, NF1, PHF6, PRPF8, RB1, RUNX1, SH2B3, STAG2, TET2, TP53, ZRSR2*<br><br>Hotspot regions: *ABL1, BRAF, CBL, CSF3R, DNMT3A, FLT3, GATA2, HRAS, IDH1, IDH2, JAK2, KIT, KRAS, MPL, MYD88, NPM1, NRAS, PTPN11, SETBP1, SF3B1, SRSF2, U2AF1, WT1*<br><br>Genes involved in gene fusions: *ABL1, ALK, BCL2, BRAF, CCND1, CREBBP, EGFR, ETV6, FGFR1, FGFR2, FUS, HMGA2, JAK2, KMT2A, MECOM, MET, MLLT10, MLLT3, MYBL1, MYH11, NTRK3, NUP214, PDGFRA, PDGFRB, RARA, RBM15, RUNX1, TCF3, TFE3*

Table S2: Cytogenetic abnormalities observed in 50 of 156 CMML patients.

Karyotype	*n* = 156 *n* (%)
**Normal**	106 (67.9)
**Abnormal**	50 (32.1)
+8 isolated	11
-7/del(7q) isolated	7
-Y	7
Complex karyotype	6
Others	
47,XX,+8[5]/48,idem,+21[2]	1
46,XY,t(5;6)(q31;q22);t(12;20)(p13;q11)[20]	1
46,XX,del(17)(p12)[20]	1
47,XY,+21[7]/46,XY[20]	1
46,XX,t(5;16)[3]/46,XX[17]	1
46,XY,t(3;7)(q26;q21)[10]/46,XY,idem,+13[4]	1
47,XY,+mar[2]/46,XX[30]	1
47,XY,+21[12]/46,XY[8]	1
46,XX,del(11)(q22q24)[14]/46,idem,del(12)(p12p13)[5]	1
46,XY, t(2;3) (p21;q27)[26]	1
46XY,del(11)(q14.3) [20]	1
46,XX,del(5)(q33)[5]/46,XX[15]	1
46,XY,add(6)(q23)[4]/46 XY[16]	1
46,XX,del-5(q13q33)[18]	1
46,XY,del(12)(p12)[8]/46, XY[12]	1
46,XY,del(20)(q11q13)[7]/46,XY[13]	1
47,XY,+11[12]/46,XY[8]	1
46,XY,t(14;15)(q32;q22)[14]	1
46,XY,del(20)(q11q13)[8]/46,XY[8]	1

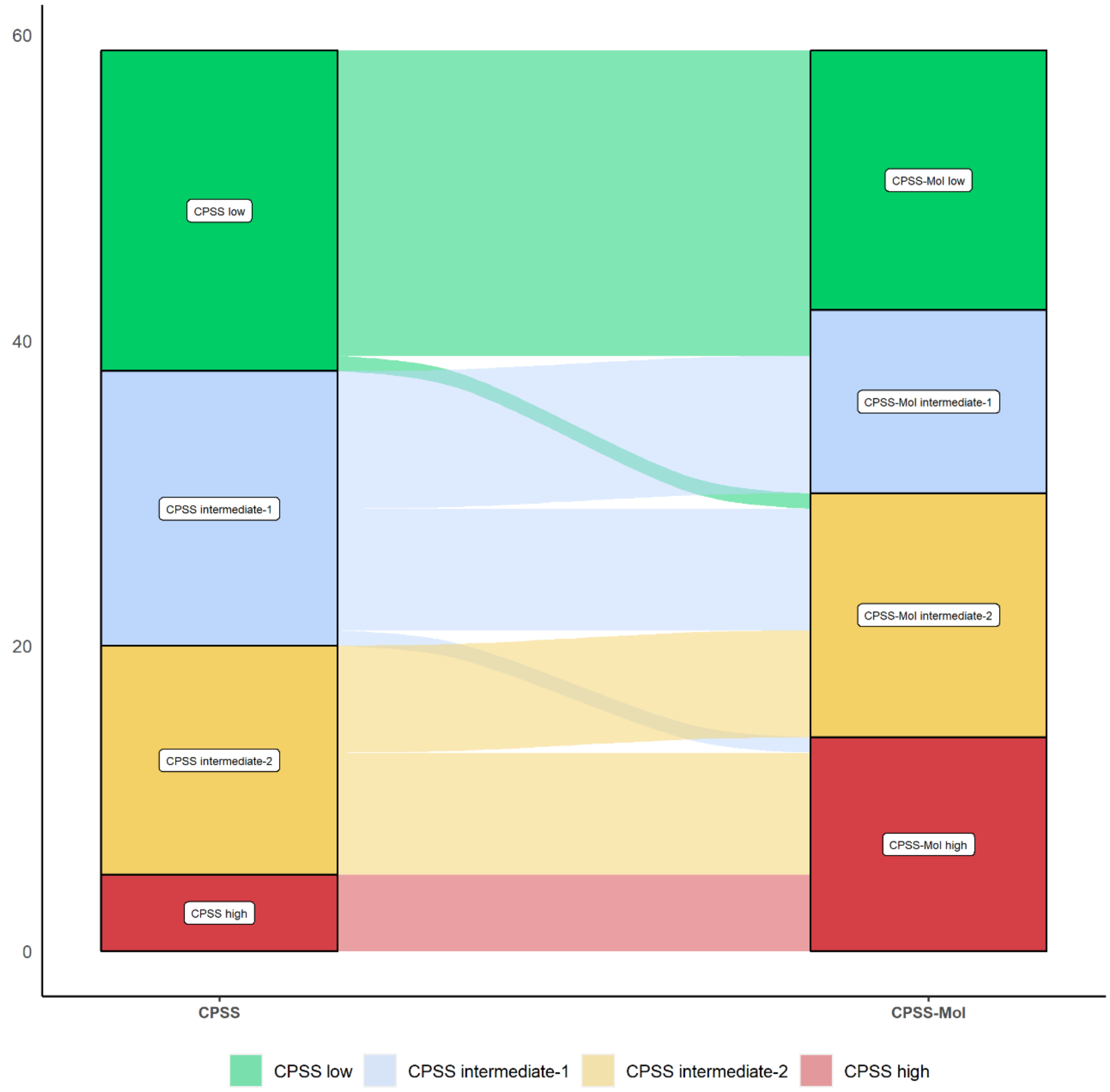

Figure S1: Alluvial plot of the patients included in the study according to CPSS and CPSS-Mol scores.

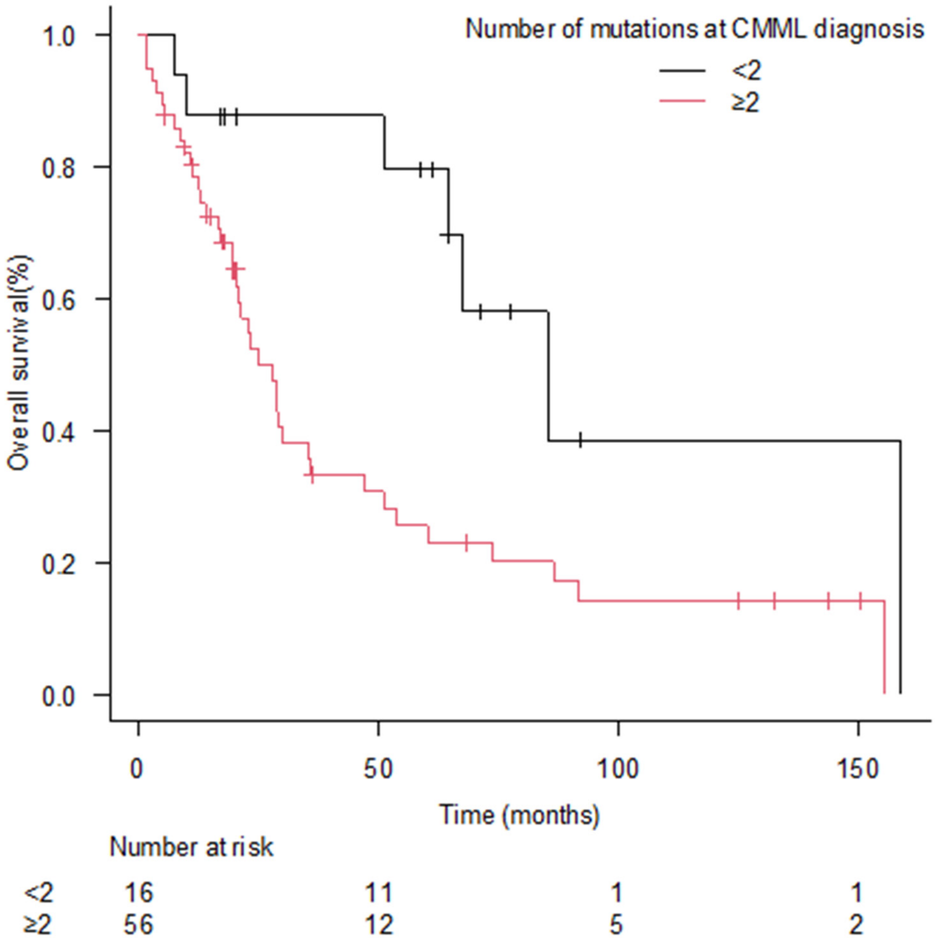

Figure S2: Overall survival according to the number of mutations detected at CMML diagnosis.

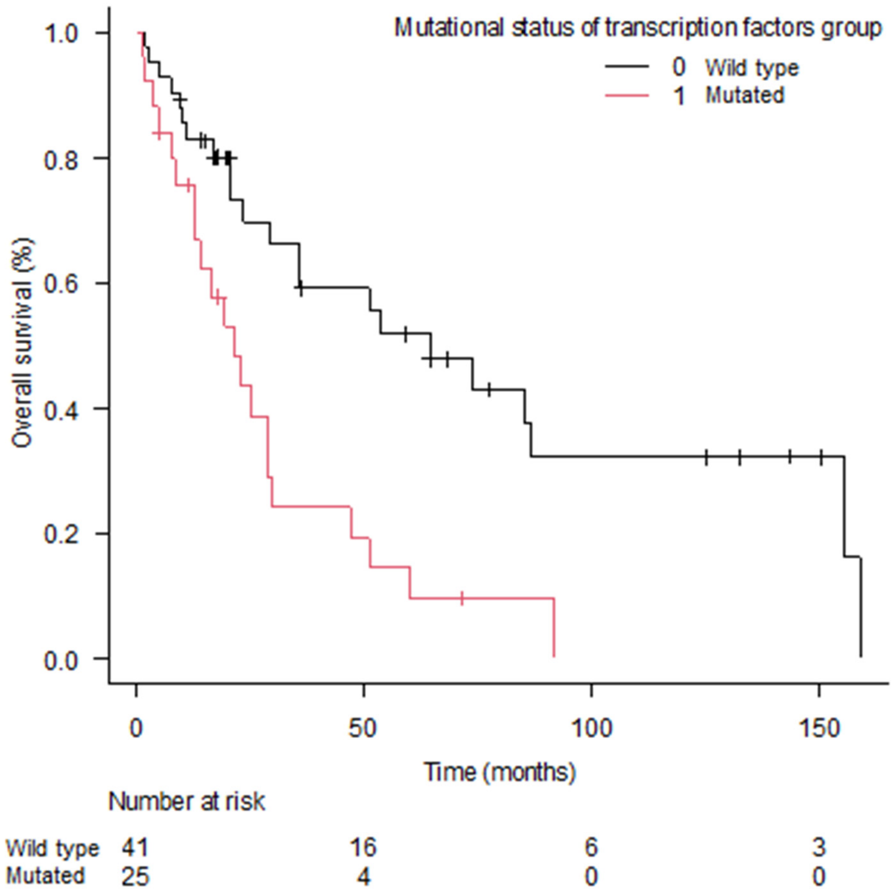

Figure S3: Overall survival according to the mutation status of transcription factors.
